# Epidemiological, etiological, and serological characteristics of hand, foot, and mouth disease in Guizhou Province, Southwest China, from 2008 to 2023

**DOI:** 10.1371/journal.pntd.0013394

**Published:** 2025-08-18

**Authors:** Fajin Li, Dan Wang, Fei Su, Suye Zhao, Jun Guo, Fumin Zhang, Xueqin Ran, Jiafu Wang, Shijun Li

**Affiliations:** 1 Key laboratory of Plant Resource Conservation and Germplasm Innovation in Mountainous Region (Ministry of Education), Collaborative Innovation Center for Mountain Ecology & Agro-Bioengineering (CICMEAB), College of Life Sciences/Institute of Agro-bioengineering, College of Animal Science, Guizhou University, Guiyang, Guizhou, China; 2 Laboratory Center, Institute of Infectious Disease Control, Guizhou Center for Disease Control and Prevention, Guiyang, Guizhou, China; University of Malaya Faculty of Medicine, MALAYSIA

## Abstract

**Background:**

Hand, foot, and mouth disease (HFMD) is caused by more than 20 different enteroviruses (EVs). The predominant EV serotypes of HFMD have been continuously changing in recent years. Guizhou Province has reported higher rates of severe and fatal cases of HFMD. However, comprehensive studies on its epidemiology, etiology, and serological characteristics have remained limited in recent years.

**Methods:**

We collected epidemiological and laboratory data from HFMD cases between 2008 and 2023, analyzing the data by age, gender, disease severity, and EV serotypes. Clinical samples from these cases were collected to isolate EVs. The VP1 gene was amplified from isolates of enterovirus A71 (EV-A71), coxsackievirus A16 (CV-A16), coxsackievirus A6 (CV-A6), and coxsackievirus A10 (CV-A10), and the sequences were analyzed. We collected 432 serum samples from healthy individuals from 2019 to 2022 to assess antibodies against CV-A6, CV-A10, CV-A16, and EV-A71 of HFMD.

**Results:**

A total of 513,143 HFMD cases were reported in Guizhou Province from 2008 to 2023, including 9052 (1.76%) severe cases and 193 (0.038%) deaths. In laboratory-confirmed cases, EV-A71 was the dominant serotype from 2008 to 2012; other EVs became predominant from 2013 to 2018, and CV-A6 predominated in 2019, 2022, and 2023. Interestingly, novel epidemiological patterns of CV-A6 infection were observed, with a high incidence every other year in various cities since 2019. Among 432 healthy individuals, the overall seroprevalence rates of CV-A6, CV-A10, CV-A16, and EV-A71 were 62.04%, 54.17%, 54.63%, and 64.35%, respectively. Additionally, over 70% of the participants had neutralizing antibodies (NtAbs) against at least two types of these enteroviruses. Phylogenetic analysis revealed that CV-A16 isolates clustered into the B1a or B1b evolutionary branches, while EV-A71, CV-A6, and CV-A10 isolates belonged to the C4a, D3a, and C subgenotypes, respectively.

**Conclusions:**

This results indicate differences in the incidence of major HFMD pathogens across years, regions, and populations. Other EVs, predominantly CV-A6, have become the main pathogens causing HFMD since 2019. CV-A6, CV-A10, CV-A16, and EV-A71 exhibited relatively high seroprevalence rates. Currently, there is an urgent need to develop multivalent vaccines and implement effective measures to reduce incidence of HFMD.

## Author summary

Hand, foot, and mouth disease (HFMD) is an infectious disease of global concern caused by a wide range of human enteroviruses (EVs). In recent years, the predominant serotypes of HFMD have been continuously changing, but only the EV-A71 vaccine for HFMD has been approved in China. In this study, we analyzed the epidemiological characteristics of 513,143 reported HFMD cases. Additionally, we collected 432 serum samples from healthy individuals to assess antibodies against coxsackievirus A6 (CV-A6), coxsackievirus A10 (CV-A10), coxsackievirus A16 (CV-A16), and enterovirus A71 (EV-A71). We identified the genotypes of the main serotypes through VP-1 sequencing. Other EVs, primarily represented by CV-A6, have become the primary pathogens of HFMD in Guizhou Province since 2019. The seroprevalence rates of CV-A6, CV-A10, CV-A16, and EV-A71 were relatively high among healthy individuals, and more than 70% of individuals had neutralizing antibodies (NtAbs) against at least two serotypes of these enteroviruses. Therefore, there is an urgent need to develop multivalent vaccines for other EVs, which could effectively target the primary pathogens associated with HFMD. Furthermore, it is essential to comprehend the epidemiology and antibody characteristics of the main serotypes associate with HFMD in order to formulate national vaccination policies for the future.

## Introduction

Hand, foot, and mouth disease (HFMD) is a major global public health issue caused by multiple human enteroviruses (EVs) [1]. This disease is prevalent among infants and young children under 5 years of age, but it can also affect adolescents and adults. HFMD in children usually manifests as fever and papulovesicular rashes on the palms [2]. The virus can also invade the respiratory system, central nervous system, cardiovascular system, leading to encephalitis, myocarditis, pulmonary edema, flaccid paralysis, and other complications. In severe cases, critically ill children may die from the disease [3].

The first reported case of HFMD was documented by researchers in Canada in 1957 [4]. The differences in serotypes of coxsackievirus group were first discovered in 1947 by Dalldorf and Sickles [5]. More than 20 different EVs have been isolated from clinical samples of HFMD patients. In the past, the predominant EV serotypes associated with HFMD were coxsackievirus A16 (CV-A16) and enterovirus A71 (EV-A71). However, this epidemiological pattern is gradually changing due to high rates of gene mutations and recombinations, as well as the launch of the EV-A71 vaccine [6]. Other EVs have replaced EV-A71 and CV-A16 as the main serotypes of HFMD in recent years. Other emerging EV strains, including coxsackievirus A6 (CV-A6) and coxsackievirus A10 (CV-A10), have been frequently reported in Finland, France, and Japan since 2010 [7–10]. CV-A6-associated HFMD outbreaks are becoming increasingly common in several regions, including Beijing, Shanghai, Tianjin, Qingdao, Guangzhou, and Nanjing [11–15]. Despite the widespread occurrence of CV-A6-driven outbreaks across China, however, the epidemiological and pathogenic characteristics of the predominant EV serotypes in Guizhou Province remain unclear.

In China, HFMD has been classified as a category C notifiable communicable disease since 2008 [16]. Currently, the only vaccine officially approved for HFMD is the EV-A71 vaccine in China. Three inactivated EV-A71 vaccines were approved and launched in 2015, January 2016, and late 2016, respectively [17–19]. Phase III clinical trials demonstrated that the three inactivated EV-A71 vaccines showed over 90% efficacy against EV-A71-associated HFMD and nearly 100% efficacy in preventing severe manifestations of the disease [17,18,20]. Regions with high population density-particularly the Shanghai-Zhejiang-Fujian and Beijing-Tianjin areas demonstrated vaccination coverage rates of 40% and 30%, respectively. This contrasted with the Southwest China area, which showed substantially lower vaccination rate at 24% [20]. Notably, EV-A71 vaccines have not yet been incorporated into the national immunization program (NIP) as a free pediatric vaccine in China. Parental willingness to vaccinate exhibits marked geographic heterogeneity, with coverage rates ranging from 3% to 40% across China by 2018 [20]. The EV-A71 vaccine was introduced in Guizhou Province in 2016, and the vaccination rate ranged from 0.96% to 9.86% in the province between 2016 and 2023.

Guizhou Province is located in the Yunnan-Guizhou Plateau region of southwest China (24.37°N–29.13°N and 103.36°E–109.35°E). It has a population of over 38 million distributed across 9 prefecture-level cities and 88 counties. The province experiences a warm and humid subtropical monsoon climate, with environmental conditions conducive to enterovirus transmission. The Guizhou Center for Disease Control and Prevention (Guizhou CDC) has established an epidemiological surveillance system and a dedicated laboratory for HFMD surveillance since 2008. Additionally, 9 municipal and 88 county-level CDCs are responsible for epidemic investigation, sample collection, and diagnostic testing.

We analyzed 513,143 reported HFMD cases and collected 432 serum samples from healthy individuals in Guizhou Province. The objectives of this study were to determine the epidemiological characteristics of HFMD, to identify predominant enterovirus serotypes, and to investigate neutralizing antibodies against these predominant serotypes. The shifts in predominant serotypes associated with HFMD have been observed in Guizhou Province since 2013. Meanwhile, monovalent EV-A71 vaccines do not offer cross-protection against other serotypes and have low coverage rates in the province. These factors may contribute to the persistent epidemic of HFMD in Guizhou and inform enhancements to pathogen surveillance for HFMD. Additionally, these findings offer essential insights for evaluating vaccine effectiveness and developing multivalent vaccines.

## Results

### The temporal, geographical distribution and population differences

A total of 513,143 HFMD cases were reported based on clinical symptoms in Guizhou Province between 2008 and 2023. The average annual number of cases was 32,0711, with an average annual incidence rate of 88.94 per 100,000 population, including 9,052 severe cases (1.76%) and 193 fatal cases (0.038%) (S1 Table).

**Fig 1:**
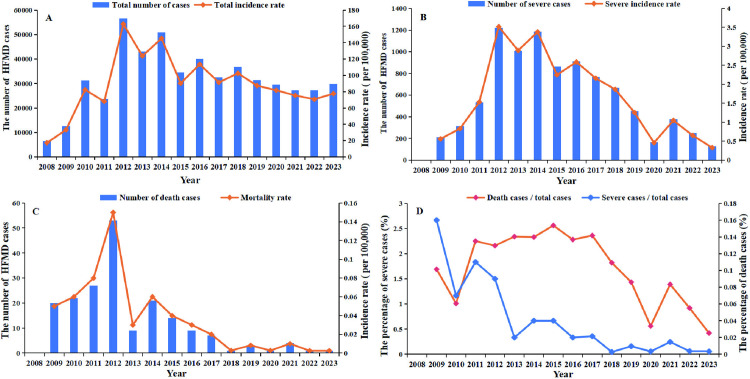
The annual distribution of HFMD cases by severity in Guizhou, China, from 2008 to 2023. (A) Based on all cases of HFMD. (B) Based on mild cases of HFMD. (C) Based on severe cases of HFMD. (D) The percentage of severe and death cases.

In Guizhou Province, HFMD exhibited year-round transmission, with the total reported cases showing a fluctuating downward trend (Fig 1A). From 2008 to 2010, case numbers increased markedly. Notably, a biennial peak pattern emerged after 2010, with even-numbered years (e.g., 2010, 2012, 2014, 2016, 2018) consistently exceeding case counts from preceding odd-numbered years. COVID-19 containment measures implemented may be associated with a gradual decline in cases between 2020 and 2022, although there was a resurgence in 2023 (Fig 1A). The number and rate of severe cases peaked in 2012, followed by a fluctuating downward trend (Fig 1B). Mortality rates paralleled this trend, decreasing sharply post–2012. Annual deaths stabilized at single-digit levels since 2016 (Fig 1C). The proportion of severe cases among total cases ranged from 0.42% to 2.56% (peaking in 2015 and reaching the lowest level in 2023), while the proportion of death cases per total cases exhibited a fluctuating downward trend, with the highest rate recorded in 2009 and the lowest in 2023 (Fig 1D).

**Fig 2:**
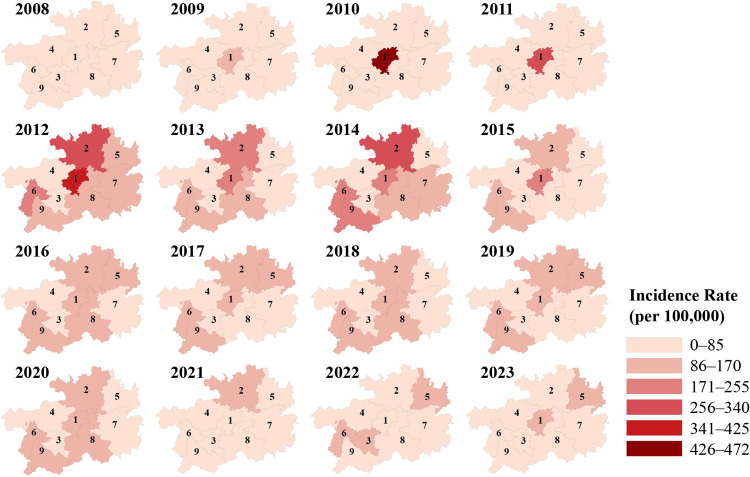
The incidence rates of HFMD across cities in Guizhou Province, China, from 2008 to 2023. The numbers represent the following cities: 1. Guiyang, 2. Zunyi, 3. Anshun, 4. Bijie, 5. Tongren, 6. Liupanshui, 7. Qiandongnan, 8. Qiannan, 9. Qianxinan. The figure was created using QGIS software (https://www.qgis.org/). The base layers of the map were extracted by the National Platform for Common GeoSpatial Information Services: https://cloudcenter.tianditu.gov.cn/administrativeDivision.

During the period 2008–2023, the incidence rates of HFMD across cities in Guizhou Province ranged from 0.42 to 471.86 per 100,000 population (Fig 2). The lowest incidence was observed in Qianxinan Prefecture (0.42/100,000), whereas the peak incidence occurred in Guiyang City (471.86/100,000). During 2010–2011, elevated HFMD incidence was predominantly clustered in central regions, particularly in the provincial capital Guiyang. The three-year average incidence rate reached a historical peak of 136.55/100,000 between 2012 and 2014, with high-incidence areas expanding progressively to northern and western regions. The city-level range of incidence rates decreased to 24.93–178.63 per 100,000 from 2015 to 2023, with the overall rate stabilizing across regions. However, localized higher incidence rates persisted in certain areas (such as Guiyang, Zunyi, and Liupanshui) (Fig 2).

**Fig 3:**
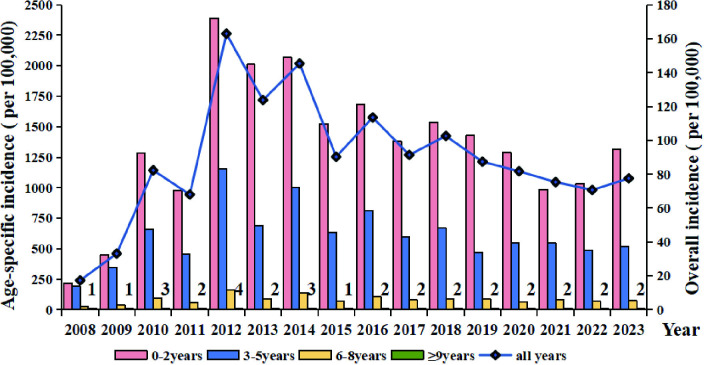
The incidence rates of HFMD across various age groups in Guizhou Province, 2008–2023.

A total of 313,344 male HFMD cases were reported in Guizhou Province from 2008 to 2023, with an annual incidence rate of 139 per 100,000 population. Female cases numbered 200,328, corresponding to an annual incidence rate of 92 per 100,000 population. The male-to-female incidence ratio was 1.51:1, with a significantly higher incidence rate in males compared to females (χ^2^ = 23.651, P < 0.0001) (Fig 3, S1 Table). Overall, the incidence rate exhibited a declining trend with increasing age. From 2008 to 2023, children aged 0–5 years accounted for 98.12% of the reported cases, with an average annual incidence rate of 979.62 per 100,000. The highest incidence was observed in the 0–2–year age group (1347.70 per 100,000 population annually), followed by the 3–5–year age group (611.54 per 100,000 population annually) (Fig 3, S1 Table).

### Novel aetiology changes of HFMD cases

**Fig 4:**
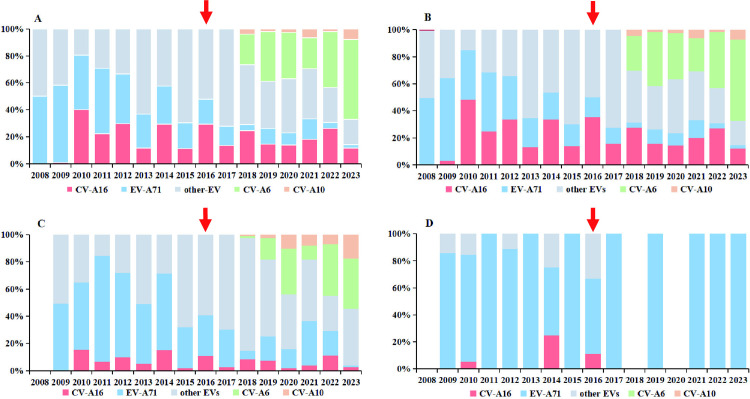
The proportion of enterovirus serotypes in HFMD cases by severity in Guizhou, China, 2008–2023. (A) Based on all cases of HFMD. (B) Based on mild cases of HFMD. (C) Based on severe cases of HFMD. (D) Based on fatal cases of HFMD. The arrow indicates the year when the EV-A71 vaccine was introduced in Guizhou Province.

In Guizhou province, a total of 513,143 HFMD cases were reported to the surveillance system from 2008 to 2023, of which 74,030 (14.43%) cases were confirmed by laboratory testing. The result showed that 39,144 (52.88%) cases were confirmed as EV-positive by laboratory testing, including 33,871 (86.53%) mild cases, 5,119 (13.08%) severe cases, and 154 (0.39%) fatal cases. Among these, 7,233 (18.48%), 8,126 (20.76%), 6,615 (16.90%), 744 (1.90%), and 16,548 (42.27%) were associated with EV-A71, CV-A16, CV-A6, CV-A10, and other EVs, respectively (Fig 4).

The predominant EV serotypes associated with HFMD have shifted continuously in recent years. From 2008 to 2012, EV-A71 was the dominant serotype among total HFMD cases, accounting for 55.56%, 56.98%, 40.44%, 48.65%, and 37.29%, respectively. Subsequently, other EVs became predominant from 2013 to 2018, with annual proportions of 62.85%, 42.28%, 69.19%, 51.48%, 72.08%, and 44.46%, respectively. CV-A6 emerged as the dominant serotype in 2019, 2022, and 2023, accounting for 36.94%, 41.21%, and 59.20%, respectively (Fig 4A). The serotype distribution pattern in mild cases corresponded closely to that of total cases throughout the study period (2008–2023) (Fig 4B). Among 5,119 severe cases, other EVs were the primary causative pathogens (48.33%) (Fig 4C). Notably, EV-A71 was responsible for 85.07% of the 154 fatal cases (Fig 4D). Significant annual variations were observed in the number of severe cases caused by other EVs (χ^2^ = 15.47, P < 0.001). Furthermore, EV positivity rates exhibited a hierarchical trend: highest in fatal cases, intermediate in severe cases, and lowest in mild cases (P < 0.01).

**Fig 5:**
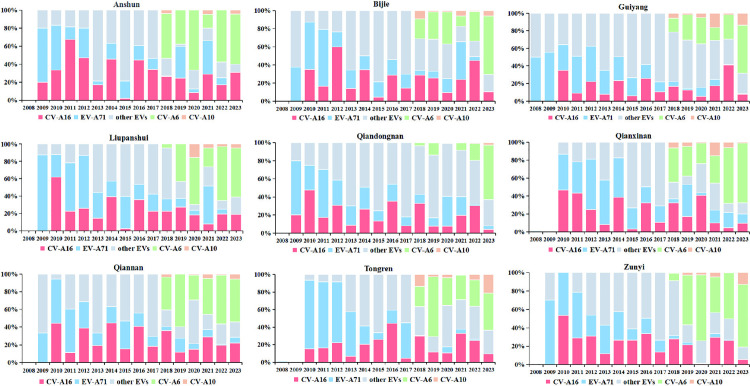
The proportion of EV serotypes in confirmed HFMD cases by city in Guizhou, China, 2008–2023.

Temporal and spatial variations in HFMD-associated pathogen composition were analyzed across cities in Guizhou Province from 2008 to 2023. The analysis revealed that CV-A6 exhibited a fluctuating yet progressive increase since 2019, ultimately emerging as the predominant serotype in all surveyed cities by 2023 (Fig 5). Notably, CV-A6 demonstrated a biennial epidemic pattern since 2019, with alternating peaks across geographic regions. Specifically, cities including Anshun, Bijie, Guiyang, Liupanshui, and Zunyi showed higher proportions during even-numbered years, whereas Qiandongnan, Qiannan, Qianxinan, and Tongren exhibited elevated proportions in odd-numbered years (Fig 5). These findings highlight the necessity of prioritizing CV-A6 surveillance in HFMD surveillance systems and vaccine development strategies, due to its significant influence on regional disease patterns and control efficacy.

**Fig 6:**
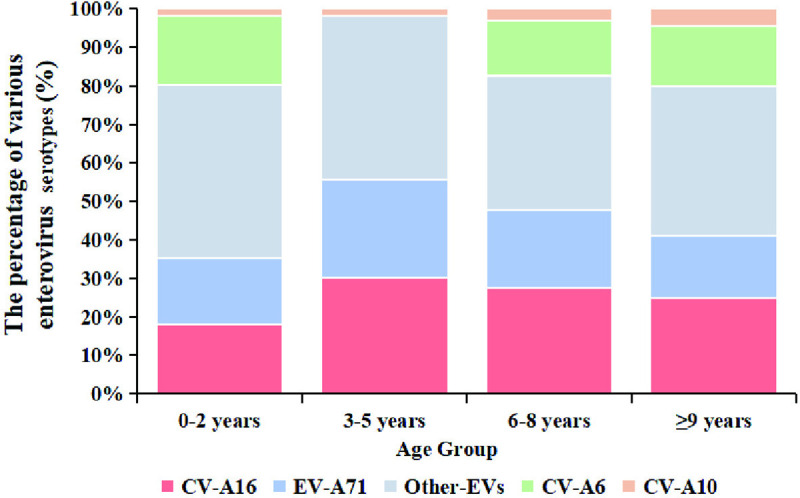
The positive rates of EV across various age groups in Guizhou Province, China, 2008–2023.

Across all age groups, other EVs constituted the predominant HFMD-associated serotypes (Fig 6). The highest infection rate of other EVs was observed in the 0–2 years age group, representing 46.81% of all EV serotypes. Notably, CV-A6 emerged as the predominant serotype within other EVs, accounting for 14.41–17.88% of total EV serotypes across all age groups. In contrast, CV-A10 exhibited significantly lower infection rates (1.57–4.5%) than other serotypes in all age groups (P < 0.05) (Fig 6).

### The seroprevalence and GMTs of CV-A6, CV-A10, CV-A16, and EV-A71

**Fig 7:**
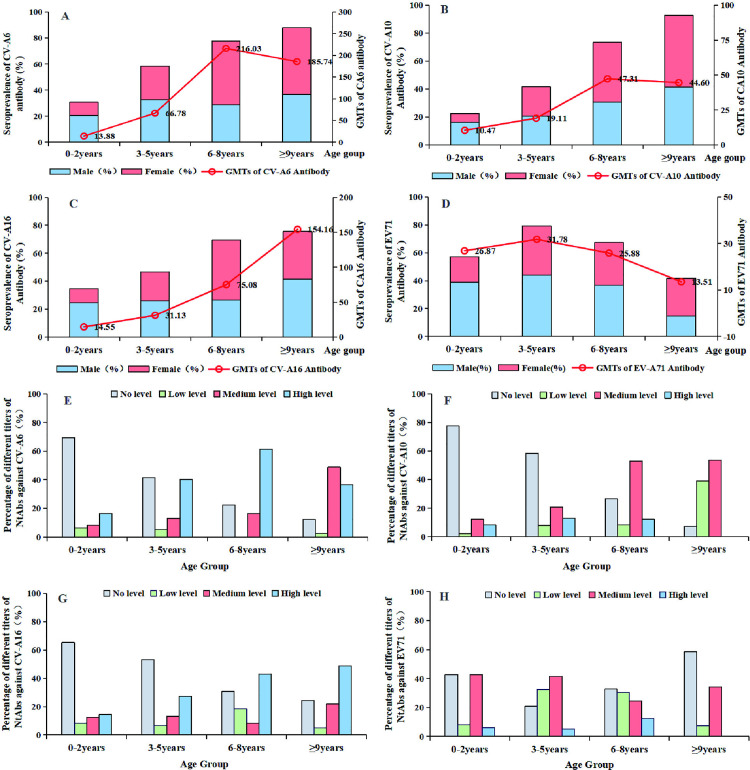
Seroprevalence and GMTs of NtAbs against CV-A6, CV-A10, CV-A16, and EV-A71 by age group in Guizhou Province.

A total of 432 healthy individuals were tested for NtAbs against CV-A6, CV-A10, CV-A16, and EV-A71. The seropositive rates of CV-A6, CV-A10, CV-A16, and EV-A71 were 62.04% (95% CI: 57.5–66.6%), 54.17% (95% CI: 49.5–58.9%), 54.63% (95% CI: 49.9–59.3%), and 64.35% (95% CI: 59.8–68.9%), respectively, which showed significant differences (χ^2^ = 14.3, P < 0.005). Of these, more than 90% of healthy individuals carried positive NtAbs against at least one serotype of the four enteroviruses, and over 70% of the participants had positive NtAbs against at least two types of these enteroviruses (Table 1). Four age groups were divided into 0–2 years, 3–5 years, 6–8 years, 9–17 years. Among them, 47.62% (120/252), 34.13% (86/252), 42.06% (106/252), 70.63% (178/252) of children aged ≤ 5 years had NtAbs against CV-A6, CV-A10, CV-A16, and EV-A71, respectively (Table 1). Furthermore, the seroprevalence rates of CV-A6, CV-A10, and CV-A16 were lowest in the 0–2 year age group, reaching the highest levels in the 9–17 age group (Table 1, Fig 7A–7C). The seroprevalence rate of EV-A71 was higher in the 0–2–year–old group (57.14%) compared to other serotypes, and peaked in the 3–5–year–old group (79.22%). The seroprevalence in females was significantly higher than in males among subjects seropositive for CV-A6 and CV-A10 (P < 0.05), whereas no significant differences were observed in EV-A71 and CV-A16 seroprevalence (Table 1, Fig 7A–7B).

**Table 1:** The demographic characteristics and Seroprevalence of participants in the serological study.

Age groups (years)	Total n (%)	Male n (%)	Female n (%)	Seroprevalence n (%)					
				Total	CV-A6	CV-A10	CV-A16	EV-A71	All four serotypes
0–2	98 (22.69)	64 (65.31)	34 (34.69)	82 (83.67)	30 (30.61)	22 (22.45)	34 (34.69)	56 (57.14)	4 (4.08)
3–5	154 (35.65)	86 (55.84)	68 (44.16)	148 (96.1)	90 (58.44)	64 (41.56)	72 (46.75)	122 (79.22)	30 (19.48)
6–8	98 (22.69)	40 (40.82)	58 (59.18)	96 (97.96)	76 (77.55)	72 (73.47)	68 (69.39)	66 (67.35)	32 (32.65)
≥ 9	82 (18.98)	36 (43.90)	46 (56.10)	82 (100)	72 (87.80)	76 (92.68)	62 (75.61)	34 (41.46)	18 (21.95)
Total	432	226 (52.31)	206 (47.69)	408 (94.44)	268 (62.04)	234 (54.17)	236 (54.63)	278 (64.35)	84 (19.44)

The GMTs of the four serotypes showed statistically significant differences across various age groups (χ^2^ = 14.3, P < 0.05). The GMTs of CV-A6, CV-A10, and CV-A16 were lowest in the 0–2–year–old age group and increased with age; however, the GMTs of EV-A71 peaked in the 3–5–year–old group and decreased with age (Fig 7A–7D). To further analyze the antibody titres, four NtAb titre ranges were categorized: no level (< 8), low level (≥ 8 and < 64), medium level (64 – 256), and high level (≥ 512) [21]. Our results showed that the titre distributions of NtAbs against the four viruses among the different age groups were diverse. The proportions of high titres for the four serotypes increased with age and reached a higher level in the 6–8–year–old age group (Fig 7E–7H).

### Phylogenetic analysis of the VP1 region of CV-A6, EV-A71, CV-A16, and CV-A10

A total of 20 CV-A6 isolates, 20 EV-A71 isolates, 20 CV-A16 isolates, and 20 CV-A10 isolates in this study were obtained from Guizhou Province (S2 Table). We prioritized sequencing of strains that were collected within 48 hours and exhibited significant enterovirus-specific CPE. Additionally, the sampling locations for these strains should cover as many cities as possible. The reference sequences were chosen to encompass the sequences covering a wider range of locations, countries, provinces, and time periods, while sequences with high homology or significant errors were excluded.

**Fig 8:**
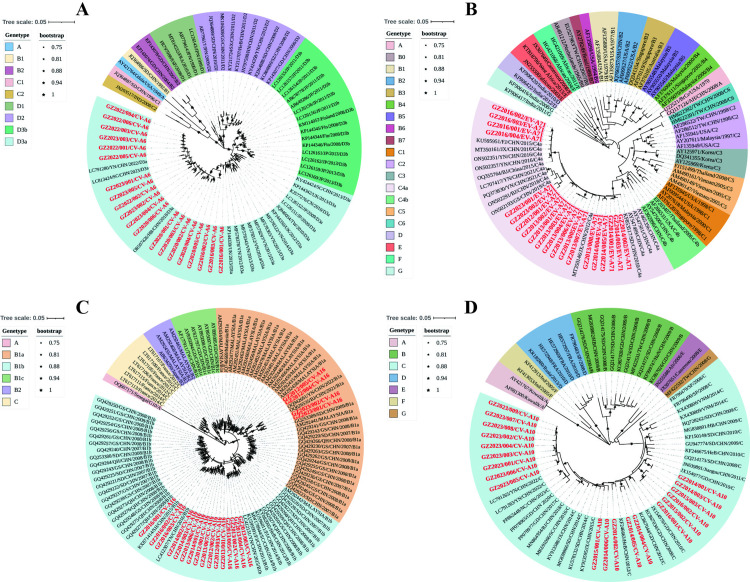
Molecular Characterization and Phylogenetic Analysis of HFMD Strains from VP1 Gene in Guizhou, China 2008-2023. The maximum likelihood (ML) tree was built based on the VP1 coding region, and used to identify the genotype of the strains obtained in this research. The VP1 sequences identified in this study are marked with red. (A) Molecular typing of 20 CV-A6 strains. (B) Molecular typing of 20 EV-A71 strains. (C) Molecular typing of 20 CV-A16 strains. (D) Molecular typing of 20 CV-A10 strains.

A total of 72 sequences were used to construct the CV-A6 phylogenetic tree. These include 20 VP1 sequences of CV-A6 from this study and 52 international CV-A6 sequences obtained from GenBank, which also include the Gdula prototype strain. According to the principle of EV genotype division, with VP1 gene identity greater than 85%, CV-A6 can be classified into four genotypes: A to D [22–24]. The D genotype is the largest cluster and can be further divided into three subtypes: D1, D2, and D3 [25–27]. The nucleotide identities of CV-A6 strains between the Guizhou isolates and the representative strains of the D3 subgenotype were 86.6–95.4%, while the amino acid identities were 93.4–96%. Currently, the D3a subgenotype of the CV-A6 is the predominant subgenotype in China [25,27]. The 20 Guizhou CV-A6 isolates were all found to be located on the D3a branch, and were closely related to the strains from Sichuan, Hebei, and Yunnan provinces of China. This suggests that CV-A6 from Guizhou Province co-circulated with CV-A6 from other regions of China (Fig 8A).

The phylogenetic analysis of EV-A71 VP1 sequences included 20 strains from Guizhou Province and 67 strains from global locations, covering China and 12 other countries. EV-A71 has been classified into seven genotypes (A to G) and fourteen genetic subtypes [28–29]. The nucleotide identities of the Guizhou EV-A71 isolates and the representative strains of the C4 genotype were 89.3–99.5%, and the amino acid identities were 96.2–100%. All EV-A71 isolates in this study clustered into genotype C4, specifically within the C4a evolutionary branches of the evolutionary tree. Additionally, these isolates exhibited a high level of nucleotide and amino acid homology with the representative strains of the C4a subtype of EV-A71 (Fig 8B).

The phylogenetic tree was constructed using 20 CV-A16 VP1 sequences from this study and 92 global CV-A16 reference sequences. The CV-A16 strains are classified into the A, B, and C genotypes [30]. Genotype B is divided into B1 and B2 subgenotypes. Additionally, B1 subgenotype is categorized into the evolutionary branches Bla, Blb, and Blc [31–32]. The nucleotide identity of CV-A16 isolates in Guizhou and representative strains B1a and B1b were 87.2–97.1% and 87.5–99.6%, while the amino acid identity were 97.9–100% and 97.9–100%, respectively. The results indicated that all CV-A16 isolates in this study were grouped into B1 genotype. Among these, four strains were identified as the Bla subgenotype, while sixteen strains were classified as the B1b subgenotype. A transmission chain of the Blb subgenotype circulated from 2012 to 2016, and another chain of the Bla subgenotype emerged in 2012 and 2023 (Fig 8C).

The phylogenetic analysis incorporated 20 entire CV-A10 VP1 sequences from Guizhou Province and 49 globally representative CV-A10 strains, including isolates from China and eight other countries. The CV-A10 strain is divided into seven subgenotypes, A-G [33–36]. The evolutionary tree indicated that the Guizhou CV-A10 isolates were clustered within genotype C in China, closely related to strains from Yunnan, Guangdong, Sichuan, Shandong, and Jiangsu provinces. Therefore, these Guizhou isolates showed a high homology with representative strains and were classified within the same evolutionary branch (Fig 8D).

## Discussion

According to data from the World Health Organization (WHO), a high incidence of HFMD was observed in China [37–39]. HFMD has been classified as a Class C statutory notifiable infectious disease in China since 2008 [40]. The incidence rate of HFMD in China ranks first among Class A, B, and C infectious diseases, with severe cases remaining consistently high [41–42]. The national incidence of HFMD in China from 2008 to 2023 has been reported as 122.36 per 100,000 population according to the National Notifiable Disease Surveillance Reporting System of China. Our study found that the incidence of HFMD in Guizhou was 88.94 per 100,000 population from 2008 to 2023. The average severe case rate of HFMD in Guizhou Province was 1.76% from 2008 to 2023, which was higher than the national average of 0.69%. The average mortality rate in Guizhou was 0.038%, which was higher than the national average of 0.014% from 2008 to 2023. These results indicated that Guizhou has a higher rate of severe cases and mortality. The number of HFMD cases has consistently ranked among the top three of all notifiable infectious diseases in Guizhou Province since 2009 [11]. This study presents the pathogens changes, molecular epidemiology, and serological features of HFMD in Guizhou Province from 2008 to 2023, based on data collected from over 0.51 million reported cases. This dataset provides the latest insights into the changes in pathogens associated with HFMD in southwestern China.

Research on the changes in EVs serotypes associated with HFMD was relatively limited in Guizhou Province; however, understanding these changes is crucial for improving immunization strategies. EV-A71 and CV-A16 were identified as the predominant pathogens of HFMD from 2008 to 2012, while other EV serotypes predominated from 2013 to 2018. This observation aligns with trends noted in other regions of mainland China [43]. In this study, CV-A6 has emerged as the predominant serotype in 2019, 2022, and 2023.This shift aligned with findings from several other regions in recent years, including Yunnan Province, Sichuan Province, and other areas in China [44–45]. CV-A6 has rapidly caused numerous global epidemics over the past decade and has been the primary pathogen causing HFMD in mainland China since 2013 [25,37]. The increasing infection rate of CV-A6 can be attributed to several factors. Firstly, the long-term circulation of EV-A71 and CV-A16 in the population may have led to natural immunity against these two serotypes [46]. The implementation of the EV-A71 vaccine has resulted in a significant reduction of over 90% in EV-A71-associated HFMD cases in China since 2016 [47]. However, the antibodies induced by EV-A71 and CV-A16 do not provide protection for susceptible populations against infections caused by other EVs serotypes, which may exert selective pressure on infections from other EVs serotypes [46]. Secondly, recombination has long been regarded as a driving force in the evolution of EVs, potentially leading to the emergence of new recombinants with increased virulence and transmissibility [48]. Additionally, this process may facilitate EVs in acquiring advantageous traits from diverse genomes throughout their evolutionary journey [37]. The novel recombinant strain of CV-A6 was identified during HFMD outbreaks in locations such as Shanghai, resulting in a more widespread rash [49]. Frequent recombination events in the non-capsid region of CV-A6 indicate that it is undergoing active evolution, while the EV-A71 subtypes has nearly ceased recombination with the other EV-A genes since their emergence. In contrast to EV-A71, the recombination of non-capsid polymorphism within the CV-A6 subtype may explain its rapid spread in a short time frame [37]. This indicates that particular attention should be paid to the serotypes changes of other EVs, especially CV-A6. Further research, including whole-genome sequencing of circulating CV-A6 strain, was necessary to ascertain whether the variations influence the epidemic incidence of CV-A6.

Seasonal circulation patterns revealed that two peaks of incidence were observed annually in this province, which was consistent with the seasonal patterns reported in regions such as Sichuan, Yunnan, and Xinjiang [50–53]. Summer has been identified as the peak season for HFMD outbreaks and represents a critical period for prevention and control measures [54]. The analysis of the age distribution indicated that the highest incidence of HFMD occurred among children aged 0–2 years in this study, which was generally consistent with the situation in mainland China [55]. In both severe and mild cases, other EVs strains were identified as the primary pathogens. Consequently, it is essential to enhance the genetic recombination and evolutionary research of other EVs to effectively monitor the epidemic situation of HFMD.

The initial investigation aimed to assess the seroprevalence and GMT levels of NtAbs against these four viruses in healthy populations in Guizhou Province during the past years. More than 90% of healthy individuals carried protective NtAbs against at least one serotype of these EVs. This indicates that these viruses were prevalent within the study population, aligning with previous research findings [46,56–59]. Among them, those aged 0–2 years had the lowest seropositivity rates and GMTs for NtAbs against CV-A6, CV-A10, and CV-A16, which is consistent with previous reports [46,56]. Therefore, preschool children under 3 years of age are more susceptible to infections caused by these three viruses. This observation aligns with the overall epidemiological trend, where the highest incidence of HFMD was reported in the 0–2–year–old age group in Guizhou Province. Additionally, the seroprevalence rates and GTMs of EV-A71 increased with age, peaking in the 3–5 year age group. EV-A71 has been the predominant pathogens of HFMD in mainland China from 2008 to 2012 [55]. The detection rate of EV-A71 has significantly decreased in Guizhou when the emergence of non-EV-A71 and non-CV-A16 EVs since 2018, alongside the introduction of the EV-A71 vaccine. Despite the introduction of the EV-A71 vaccine, this vaccine lacks cross-protection against other serotypes, and the vaccination rate in Guizhou Province remains low; therefore, the overall risk of HFMD has not been effectively controlled. The seroprevalence rats of EV-A71 in the 0–2 age group and the 3–5 age group were higher than those of other serotypes within the same groups. This phenomenon may be attributed to EV-A71 natural immunity and vaccine-induced protection, which could establish an immunological barrier in the 0–5–year–old susceptible population. As a result, this may lead to a decline in the seroprevalence and detection rates of EV-A71 in the 6–8 and ≥ 9 age groups. Vaccination is the most effective preventive measure for young children, with the optimal age range for vaccination being 6–12 months.

In this study, more than 70% of individuals showed the presence of at least two serotypes among the four viruses. Previous research has demonstrated that specific NtAbs against EVs do not offer cross-protection to other serotypes [55]. Therefore, the presence of NtAbs against multiple EVs may stem from previous infections or vaccinations. In recent years, surveillance data has shown that other EVs frequently co-circulated and infected in various combinations during HFMD outbreaks [60]. Some studies indicated that infections with multiple serotypes could exacerbate the severity of clinical symptoms, and co-circulation of different serotypes may lead to an increased frequency of genetic recombination [61]. The persistence of NtAb against the four viruses throughout life remains uncertain; however, the epidemiological profile of HFMD suggests that older children and adults are generally immune to clinical manifestations of the disease. This phenomenon is usually attributed to the gradual maturation of the immune system in older children.

This study reported on the phylogenetic characteristics of CV-A6, EV-A71, CV-A16, and CV-A10. CV-A6 has rapidly emerged as a cause of persistent global epidemics over the last decade [2,7,62]. CV-A6 outbreaks reported worldwide have predominantly been the D3 genesubtype since 2008 [25,27,37]. The 20 VP-1 sequences of CV-A6 in this study all belonged to the D3a subgenotype. This D3 cluster included isolates from Japan, Europe and China, specifically from Yunnan, Sichuan, and Hebei provinces. This suggested that CV-A6 within the D3a subgenotype has emerged as a predominant serotype in Guizhou region. The D3 isolates in this study were closely related to strains from United Kingdom in 2012 and 2013, Japan in 2009. However, the Guizhou D3 isolates formed a distinct branch separate from strains isolated in other countries. This indicated that the D3a strains in this study may have originated from mainland China, as documented in previous studies [25,37]. This finding implied that the source of Guizhou CV-A6 was relatively homogeneous with minimal variations, co-circulating with CV-A6 from other regions of China. There were three distinct periods of prevalence for CV-A6 in China, with varying genotypes identified during each epidemic phase. The complex evolution of the virus has increased the transmission speed and infectivity of CV-A6, which may also be an important reason for the continuous prevalence of CV-A6 D3 subtypes in China since 2013 [22,25,37].

The etiological analysis indicated that EV-A71 remains the primary serotype responsible for fatal cases of HFMD. EV-A71 has been classified into seven genotypes (A-G) and 14 genetic subtypes [8]. The C4 subgenotype has been circulating and prevalent in China for 20 years, consisting of two evolutionary branches, C4b and C4a [10]. Genotypes B and C of EV-A71 are the most prevalent globally. Most HFMD epidemics associated with EV-A71 were linked to either genotype B or C, or a combination of both [5]. In some outbreaks, the concurrent circulation of two or more subgenotypes has resulted in genetic recombination, leading to the emergence of new genotypes and subsequent new outbreaks. The recombination of genotype C2 of EV-A71 with CV-A8 resulted in the appearance of genotype B4, which was prevalent in Japan, Taiwan, and other countries [63–64]. Sub-genotype C1 was prevalent in Europe before 2005 but was replaced by genotype C2 after 2007 [65]. The genotype C4a of EV-A71 has been the most frequently detected sub-genotype in China to date [10]. In recent years, the evolution of the EV-A71 prevalent strain in mainland China has shifted from the C4b branch to the C4a branch, leading to an increase in transmissibility of EV-A71 [6]. In this study, the 20 EV-A71 isolates in Guizhou Province clustered into genotype C4, and belonged to the C4a evolutionary branches. Therefore, the Guizhou isolates were phylogenetically closely related to the C4 genotype strains from mainland China, indicating their co-circulation and co-evolution within the region. CV-A16 was classified into genotypes A, B, and C [21]. The results indicated that the 20 CV-A16 isolates from Guizhou Province were clustered into genotype B1, with the evolutionary branches of B1a and B1b. A B1a transmission chain emerged in 2012, followed by a new B1a transmission chain in 2023, which was closely associated with the epidemic strains in Malaysla and China. The B1a strains identified in Guizhou Province were probably introduced from other regions of China or other countries in Southeast Asia. The remaining 16 B1b strains were phylogenetically closely related to strains from Yunnan, Shanghai, Qinghai, and other provinces in mainland China. Currently, the two evolutionary branches were co-circulating in Guizhou Province. Future surveillance is required to determine whether these lineages will continue to coexist or if one evolutionary branche will eventually dominate, leading to the displacement of the other. The CV-A10 strain can be divided into seven subgenotypes: A to G [33–36]. Genotype B was prevalent in China until 2008. Subsequently, genotype C emerged. This genotype was identified in Spain and France during this period [5]. The CV-A10 isolates from Guizhou were classified as genotype C, indicating a close phylogenetic relationship with domestic strains. This finding suggestde that Guizhou strains co-circulated and evolved with other strains in mainland China.

We conducted a retrospective study on the epidemiology, etiology, and serotypes of HFMD in Guizhou Province, Southwest China, over the past 16 years. The results indicated higher seroprevalence rates of CV-A6, CV-A10, CV-A16, and EV-A71 among healthy individuals in the region. Similar trends have been observed in healthy individuals across many countries during major epidemic periods. The seroprevalence of NtAbs requires further research to assess its reliability in predicting future disease outbreaks. Other EVs, specifically CV-A6 and CV-A10, have become the predominant serotypes causing HFMD in different regions of China. Therefore, future attention should focus on surveillance the epidemiology and seroprevalence of other EVs, particularly CV-A6 and CV-A10. This will serve as a vital guide for the future development of immunization programs and control measures against HFMD. Furthermore, the long-term spread and circulation of other EVs within the population may result in the emergence of new genotypes or sub-genotypes or gradual disappearance of existing genotypes [66]. This process can lead to variation and evolution of these, thereby altering the virulence and transmissibility of other EVs. Therefore, real-time surveillance of genotype and subgenotype variations of other EVs holds scientific importance [67–68].

## Conclusion

This results indicated differences in the incidence of major pathogens associated with HFMD based on different years, regions and populations. Other EVs predominantly represented by CV-A6 have become the main pathogens causing HFMD in Guizhou Province since 2019. The seroprevalence rates of CV-A6, CV-A10, CV-A16, and EV-A71 were relatively high among healthy individuals; over 70% of the participants had positive NtAbs against at least two serotype of these enteroviruses. EV-A71, CV-A16, CV-A6 and CV-A10 isolates belonged to the C4, B1, D3, and C genotype, respectively. Therefore, there is an urgent need to research and develop multi-valent vaccines for other EVs, which could target the major pathogens associated with HFMD in the southwest region of China. In conclusion, implementing appropriate preventive and protective measures can effectively manage the incidence of HFMD.

## Materials and methods

### Ethics statement

The study protocol was approved by the Ethics Review Committee of the Guizhou University Medical College (HMEE-GZU-2024-T026) and Guizhou Center for Disease Control and Prevention (Q2024-13). All procedures complied with the approved guidelines. Written consent for clinical specimens was obtained from parents and/or guardians of participating children in the study. All personally identifiable information associated with clinical samples was anonymized to protect participants’ privacy. No adverse reactions and hazards were found in this research. Data analysis was performed at an aggregate level, with all personally identifiable information irreversibly anonymized to protect participant confidentiality. This study adhered to the ethical principles outlined in the Declaration of Helsinki.

### Case definition

HFMD was classified as a Category C notifiable infectious disease in China in May 2008 [69]. According to the Chinese guidelines for the diagnosis and treatment of HFMD (2018 edition) [70], clinical symptoms of suspected cases include: (1) maculopapular rashes or vesicles on the hands, feet, oral mucosa, or buttocks, and (2) scattered herpes in the pharyngeal isthmus, with or without fever. Confrmed cases are identified through the reverse transcription polymerase chain reaction (RT-PCR), real-time RT-PCR, or viral isolation for detecting EVs infections (such as EV-A71, CV-A16, or other non-EV-A71 and non-CV-A16 EVs) in laboratory. Cases presenting only dermal and mucosal manifestations are classified as mild cases. Patients demonstrating neurological involvement (e.g., meningoencephalitis, encephalomyelitis) and/or cardiopulmonary complications (e.g., myocarditis, pulmonary hemorrhage) are defined as severe cases, with potential for rapid progression to fatal outcomes. According to the Chinese guidelines for the diagnosis and treatment of HFMD (2018 edition) [70], the selection of clinical samples meeting HFMD case definitions for testing is based on clinical severity, epidemiological significance, and public health priorities. All severe and fatal cases must be tested. The first case in a cluster outbreak should be prioritized for testing to confirm the pathogen; subsequent cases may require targeted sampling to verify serotype consistency. Systematic sampling and testing should be conducted for mild cases in sentinel surveillance hospitals. The scope of sample testing may be expanded during HFMD outbreaks.

### Detection of viral RNA

Clinical specimens for HFMD surveillance (including throat swabs, anal swabs, stool samples, vesicular swabs, and other relevant samples) were collected through the Guizhou CDC’s routine surveillance system. Total virus RNA was extracted using the viral RNA extraction kit (TIANLONG Co., Ltd, Xi’an, China) following manufacturer protocols. EV, CV-A6, CV-A10, CV-A16, and EV-A71 were tested using commercial real-time PCR kits, including: (1) Multiplex nucleic acid detection kits for EV, EV-A71, CV-A16, CV-A6, and CV-A10 (Cat No. DY0685; Guangzhou Da An Biotechnology Co., Ltd, Guangzhou, China); (2) Pan-enterovirus nucleic acid detection kit (Cat No. YJC20101N; Bioperfectus Technologies Co., Ltd, Taizhou, China), following manufacturer protocols. Laboratory results were classified into six categories: EVs negative, EV-A71 positive, CV-A16 positive, CV-A6 positive, CV-A10 positive, or other EVs positive. From 2008 to 2012, the HFMD surveillance program included EV, EV-A71, and CV-A16. With the observed increase in non-EV-A71/CV-A16 enterovirus prevalence since 2013, tests CV-A6 and CV-A10 were integrated into the surveillance program from 2018 to 2023.

### Virus isolation and VP-1 sequencing

Clinical specimens from confirmed HFMD cases were inoculated onto human rhabdomyosarcoma (RD) cell monolayers and incubated in a 5% CO₂ incubator at 36°C for 7 days. We observed whether enterovirus-like cytopathic effect (CPE) appeared daily. Viral harvest was performed when 75–100% of the RD cells showed enterovirus-like CPE. For cultures without CPE, the cultures were harvested and seeded into a new RD cell culture for to 2–3 times. Cell cultures showing EV-like CPE were subjected to real-time RT-PCR to confirm enterovirus presence. The entire VP1 sequences of CV-A6, CV-A10, CV-A16, and EV-A71 strains were acquired using in-house primers as previously described [28,33,71,72]: CV-A6-VP1-F: 5’-CTTCGTAGTGCCACCAGATA-3’, and CV-A6-VP1-R: 5’-GTGGCGAGATGTCGGTTTA-3’; CV-A10-VP1-F: 5’-GAAACCCCTGGAGAGGCATA- 3’, and CV-A10-VP1-R: 5’-TCGTGAGCTATCTTCCCACA-3’; CV-A16-VP1-F, 5’-ATTGGTGCTCCCACTACAGC-3’, and CVA16-VP1-R: 5’-GCTGTCCTCCCACACAAGAT-3’; EV-A71-VP1-F: 5’-GCAGCCCAAAAGAACTTCAC-3’, and EV-A71-VP1-R: 5’-AAGTCGCGAGAGCTGTCTTC-3’. PCR amplicons were purified using the QIAquick Gel Extraction Kit (Qiagen, Hilden, Germany) and subsequently submitted to Sangon Biotech Co., Ltd (Chongqing, China) for DNA sequencing. The sequencing was performed bidirectionally using an automated ABI 3730 DNA sequencer, with each strand sequenced at least once.

### Test of neutralization

Neutralizing antibodies (NtAbs) were tested as previously outlined [21,73]. Serum samples from 432 healthy individuals aged betweet 5 month and 17 years (226 Males and 206 females) were obtained through Guizhou CDC’s HFMD surveillance program between 2019 and 2022. Participants who had no clinical symptoms (e.g., HFMD) or immunodeficiency disorders for at least one month before sampling were included in the study. Individuals were excluded if they had received relevant vaccines recently or were on chronic immunosuppressive therapy. A simple random sampling strategy was applied, covering both registered residents and non-registered individuals with ≥ 6 months of local residency. Samples were obtained during periods either before or after the annual HFMD incidence peak (January-February or November-December). Participants were categorized into four age groups (0–2, 3–5, 6–8, and ≥ 9 years) to assess NtAbs against CV-A6, CV-A10, CV-A16, and EV-A71 using a micro-neutralization assay. To identify the NtAbs against the four viruses, four clinical strains were used: CV-A6/Q239/GZ/CHN/2022 (D3 genotype; severe HFMD case, 2022), CV-A10/Q208/GZ/CHN/2021 (C genotype; severe case, 2021), CV-A16/Q295/GZ/CHN/2022 (B1 genotype; mild case, 2022), and EV-A71/Q459/GZ/CHN/2022 (C4 genotype; severe case, 2022). These viruses were propagated in monolayers of RD cells. After purifying the viral mixture culture through the plaque assay, the half tissue culture infective dose (TCID_50_) was determined as previously outlined [21,73]. Serum samples were inactivated at 56 °C for 30 minutes, and gradient diluted at 1:4–1:1024 (1:4, 1:16, 1:64, 1:256, and 1:1024). In 96–well microplates, we mixed 50µL of diluted serum samples with 100 TCID_50_ of virus stock (CV-A6, CV-A10, CV-A16, or EV-A71) diluted in 50µL, and incubated the mixture at 36 °C for 2 hours [21,73]. Subsequently, 1 × 10^5^ cells/ ml human RD cells were added per well. All diluted samples of serum were tested in duplicate, with human RD cell control, serum control, and virus control included on each plate. Additionally, each experiment was conducted with viral reverse titration. Plates were observed daily for EV-like CPE for 7 days (at 36 °C in a 5% CO_2_ incubator). Neutralization titers were defined as the highest serum dilution that effectively inhibiting ≥ 50% CPE of the virus-infected wells. The serum sample was considered positive with NtAb titers of ≥ 1:8 as previously reported [21]. The geometric mean titers (GMTs) were calculated and documented accordingly. GMT values were calculated and classified into four tiers as previously reported [21]: no level (< 1:8), low level (1:8 – < 1:64), medium level (1:64 – 1:256), and high level (≥ 1:512).

### Data collection and analytical methods

We obtained data on 513,143 cases of HFMD primarily from the Chinese Infectious Diseases Reporting System within the Information System of the Chinese Center for Disease Control and Prevention, covering the period from January 1, 2008, to December 31, 2023. These data mainly included fundamental information about the study subjects, such as gender, age, occupation, date of onset, time of diagnosis, case type (severe, mild, or fatal), and laboratory test results. The personal privacy components of the data were confidential; the private information and detailed address were incomplete. The demographic data on permanent residents in Guizhou Province were obtained from the Statistical Yearbook of Guizhou Province, published annually through the official website of the Guizhou Provincial Bureau of Statistics. These data provide detailed demographic characteristics, including population size, gender distribution, age structure, and other relevant details across 88 counties-all of which are essential for calculating disease incidence and related statistics.

The data were organized using Microsoft Excel 2019. The study population was categorized into four age groups: 0–2 years, 3–5 years, 6–8 years, and ≥ 9 years. Time series analysis was performed to characterize the seasonal distribution of HFMD cases and identify epidemiological peaks. We analyzed dynamic changes in disease incidence, specifically examining three dimensions: temporal distribution (monthly, seasonal, and annual patterns), geographic distribution, and demographic characteristics (gender, age, and occupation). Incidence and mortality rates were subsequently calculated. The base layers of the map were extracted by the National Platform for Common GeoSpatial Information Services: https://cloudcenter.tianditu.gov.cn/administrativeDivision. The maps were created using Quantum Geographic Information System (QGIS), an open-source software (https://www.qgis.org/). Group comparisons were analyzed using Chi-square tests or Fisher’s exact tests in SPSS17.0 (IBM Corp), with statistical significance defined as P < 0.05.

### Phylogenetic analyses

Phylogenetic trees were constructed for the relatedness analysis using Neighbor-Joining (NJ) method with the Maximum Composite Likelihood model in MEGA (version 11.0) with 1000 bootstrap replicates. The VP1 nucleotide (or deduced amino acid) sequences of the Guizhou isolates in this study and other representative strains from NCBI were pairwise aligned using MEGA (version 11.0), and the similarity matrices were analyzed with BioEdit (version 7.0.9.0). The phylogenetic trees were constructed to display the evolutionary relationships of EV-A71, CV-A16, CV-A6, and CV-A10 isolates, respectively.

## Supporting information

S1 Table Reported Cases of HFMD by Gender, Age Group, and Population in Guizhou Province, China, from 2008 to 2023. (XLSX)

S2 Table Summary of information on 80 isolates associated with HFMD cases in Guizhou Province, China, from 2008 to 2023. (XLSX)
